# Short-time weight-bearing capacity assessment for non-ambulatory patients with subacute stroke: reliability and discriminative power

**DOI:** 10.1186/s13104-015-1722-7

**Published:** 2015-11-26

**Authors:** Oliver Stoller, Heike Rosemeyer, Heiner Baur, Matthias Schindelholz, Kenneth J. Hunt, Lorenz Radlinger, Corina Schuster-Amft

**Affiliations:** Division of Mechanical Engineering, Department of Engineering and Information Technology, Institute for Rehabilitation and Performance Technology, Bern University of Applied Sciences, Pestalozzistrasse 20, 3400 Burgdorf, Switzerland; Research Department, Reha Rheinfelden, Salinenstrasse 98, 4310 Rheinfelden, Switzerland; Physiotherapy Department, Reha Rheinfelden, Salinenstrasse 98, 4310 Rheinfelden, Switzerland; Division of Health, Applied Research and Development Physiotherapy, Bern University of Applied Sciences, Murtenstrasse 10, 3008 Bern, Switzerland

**Keywords:** Cerebrovascular accident, Subacute, Rehabilitation, Motor recovery, Clinical routine

## Abstract

**Background:**

Weight-bearing capacity (WBC) on the hemiparetic leg is crucial for independent walking, and is thus an important outcome to monitor after a stroke. A specific and practical assessment in non-ambulatory patients is not available. This is of importance considering the increasing administration of high intensive gait training for the severely impaired stroke population. The aim was to develop a fast and easy-to-perform assessment for WBC on a foot pressure plate to be used in clinical routine.

**Methods:**

WBC was assessed in the frontal plane in 30 non-ambulatory patients with subacute stroke and 10 healthy controls under 3 conditions: static, dynamic, and rhythmic. Force–time curves for the hemiparetic leg (patients with stroke) and the non-dominant leg (healthy controls) were normalised as a percentage of body weight (%BW), and the means analysed over 60, 30, and 15 s (static) and the mean of the peak values for 15, 10, 5, 4, and 3 repetition trials (dynamic, rhythmic). The data were tested for discriminative power and reliability. Dynamic and rhythmic tests could discriminate between patients with stroke and healthy controls over all periods (15, 10, 5, 4, and 3 repetitions) (p < 0.001), but not the static test (60 s, p = 0.639; 30 s, p = 0.708; 15 s, p = 0.685). Excellent relative intra-session [intra-class correlation (ICC) >0.829] and inter-session reliability (ICC = 0.740) were found for 3 repetitions in the dynamic test with acceptable absolute reliability [standard error of measurement (SEM) <5 %BW, minimal detectable difference (MDD) <12.4 %BW] and no within- or between-test differences (trial 1, p = 0.792; trial 2, p = 0.067; between trials, p = 0.102).

**Conclusions:**

Three dynamic repetitions of loading the hemiparetic leg are sufficient to assess WBC in non-ambulatory patients with subacute stroke. This is an important finding regarding the implementation of a fast and easy-to-perform assessment for routine clinical usage in patients with limited standing ability.

## Background

Loading the hemiparetic leg is an important factor in gait retraining and is essential for independent walking after stroke [1]. Weight-bearing capacity (WBC) is therefore an important outcome to monitor and particularly relevant in patients with severe motor impairments who cannot walk independently, thus, are unable to perform established gait assessments.

Considering today’s capabilities in technical-assisted rehabilitation after severe stroke, i.e., the administration of high intensive gait training for the severely impaired stroke population, there is a need to assess WBC for clinical decision-making and efficacy analyses. Although previous research revealed several modalities to evaluate WBC after stroke [2–8], no rapid assessment exists to evaluate the ability to transfer load to the hemiparetic leg.

The aim was to develop and evaluate a fast and easy-to-perform assessment on a foot pressure plate to be used for clinical routine, which is of importance regarding recent advancement in gait rehabilitation of patients with severe motor impairments. This study tested static, dynamic and rhythmic single leg loading in the frontal plane for discriminative power and reliability in order to move the assessment towards practicability.

## Methods

### Participants

Thirty non-ambulatory patients with subacute stroke and 10 healthy controls participated in this study. Participants’ characteristics are shown in Table 1. Eligibility criteria for patients with stroke were: age >18 years, first-ever stroke within the last 20 weeks, able to stand without support for 60 s, no orthopaedic pathologies, functional ambulation classification <3 [9], and ability to understand the procedure. Eligibility criteria for the healthy controls were: age >18 years, able to walk without aids, no neurological or orthopaedic pathologies, and no surgery in general within the last 2 years. All participants gave signed informed consent. The ethics committee of the Swiss Canton of Aargau reviewed and approved the study (Ref. 2012/051).

**Table 1:** Participants’ characteristics

	Patients with stroke (n = 30)	Healthy controls (n = 10)	*p* value*
Men/women	19/11	4/6	
Age (years)	63.6 ± 11.6 (41–82)	59.0 ± 12.7 (32–77)	0.379
Body mass (kg)	77.0 ± 17.9 (51–123)	70.8 ± 11.4 (53–95)	0.396
Height (m)	1.7 ± 0.1 (1.6–1.9)	1.7 ± 0.1 (1.6–1.9)	0.818
Body mass index (kg/m^2^)	26.6 ± 5.7 (18.5–40.2)	24.7 ± 2.4 (19.7–27.5)	0.569
Type of stroke: ischemic/haemorrhagic	21/9		
Hemiparetic side: right/left	18/12		
Ankle splint: yes/no	4/26		
Time post-stroke (weeks)	7.4 ± 4.2 (2.1–17.7)		
Functional ambulation classification: 0–5	1.4 ± 0.7 (0–2)		

Values are given in absolute numbers (n) or mean ± standard deviation (range)

* Mann–Whitney U test

### Data collection

Participants performed two trials on separate days. A foot pressure plate (FDM-S, zebris Medical GmbH, Isny i.A., Germany; width 540 mm, length 340 mm, sampling rate 50 Hz), based on calibrated capacitive force sensors was used to measure the static and dynamic forces under each foot. Participants were placed in a standard standing position (both legs on the foot pressure plate, heels 100 mm apart, leg rotation chosen freely) within parallel bars, wearing flat closed shoes, with arms hung aside the body and eyes open. Two trained physical therapists helped the patients with stroke to get up from the wheelchair to stand directly on the foot pressure plate. All participants were allowed to hold the parallel bars during positioning of the feet, which was done by the physical therapists. The standardised parallel bars (height 100 cm) were positioned outside of the participant’s range of motion for psychological comfort and safety reasons. At any time, a trained physical therapist stood in the range for supervision or physical assistance if needed.

**Fig. 1:**
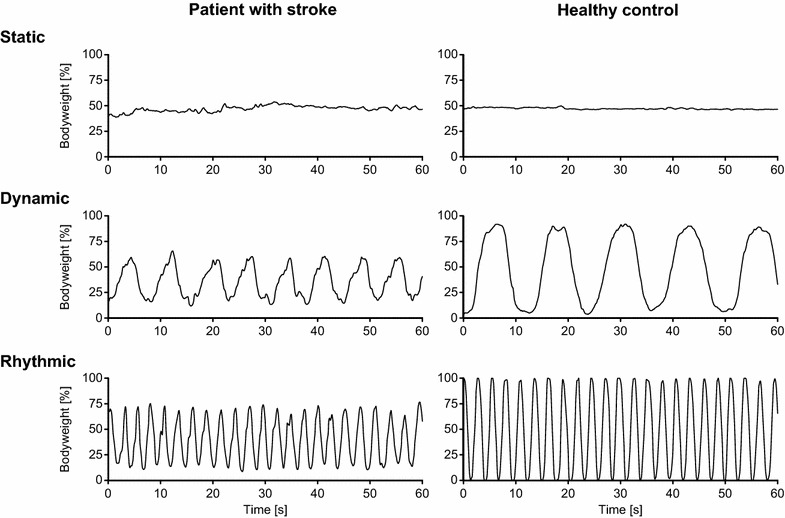
Typical test sequence. Force–time *curves* of a patient with subacute stroke (*left*) and a healthy control (*right*) during the test battery (*static, dynamic, rhythmic*)

All participants were instructed to: (1) stand as still and balanced as possible for 60 s (static); (2) alternately shift the body weight in the frontal plane as far as possible sideways at a self-selected frequency for 60 s (dynamic), and (3) alternately shift the body weight in the frontal plane as far as possible sideways at a given pace (45 beats/min controlled by a digital metronome) for 60 s (rhythmic) without holding the parallel bars (for a typical sequence please see Fig. 1). Complete foot contact to the foot pressure plate, no knee hyperextension, knee flexion <20°, and no forced hip abduction/adduction during all the tests were essential to successfully accomplish the test protocol (supervised by a trained physical therapist). There was 60 s rest in a sitting position between the 3 test modalities and exactly 48 h between the two sessions. All participants performed 1 trial in static, dynamic, and rhythmic condition per session.

### Data processing

Force–time curves for the hemiparetic leg (patients with stroke) and the non-dominant leg (healthy controls) were calculated and normalised as a %BW (non-dominant led = leg with lower  %BW in the static condition). For the static measurement, WBC was defined as the mean value of total  %BW and was calculated for 60, 30, and 15 s. WBC for the dynamic and the rhythmic modality measures was defined as the mean of the peak  %BW values (Fmax), representing the amount of single leg loading capacity. It was estimated for 15, 10, 5, 4, and 3 repetitions. Data processing was performed using customised software (ads, Version 4.01, UK Labs, Kempten, Germany).

### Statistical analysis

Differences between groups (patients with stroke vs. healthy controls) were evaluated with the Mann–Whitney U test. Intra-session and inter-session reliability were quantified using intra-class correlation coefficients (ICC_2,1_). Absolute reliability was determined by estimating the standard error of the measurement $\left[{\text{SEM}} = {\text{standard deviation of the difference }}\left( {\text{SDdiff}} \right)\sqrt { 1\,{ - }\,{\text{ICC}}}\right]$ and the minimal detectable difference (MDD = 1.96 × √2 × SEM) [10]. The Friedman test was used for intra-session comparisons of Fmax values, and the Wilcoxon test was applied for between-session comparisons of Fmax values. Two-sided p values p ≤ 0.05 were considered significant. Statistical analyses were performed using SPSS (version 20.0, IBM, Armonk NY, USA).

## Findings

WBC was significantly different between patients with stroke and healthy controls over all periods (15, 10, 5, 4, and 3 repetitions) in the dynamic test (p < 0.001) and the rhythmic test (p < 0.001), but not in the static test (60 s, p = 0.639; 30 s, p = 0.708; 15 s, p = 0.685). Further analyses in patients with stroke showed that 3 repetitions in the dynamic test (dynamic Fmax3), the fastest and easiest setup, had excellent relative intra-session [T1, ICC = 0.843 (confidence interval (CI) 95 % 0.735–0.915); T2, ICC = 0.829 (CI 95 % 0.715–0.908)] and inter-session reliability (ICC = 0.740 (CI 95 % 0.517–0.868)) with acceptable absolute reliability (SEM < 5 %BW, MDD < 12.4 %BW). No intra- and inter-session differences (T1, p = 0.792; T2, p = 0.067; T1/T2, p = 0.102) were detected (Table 2). Although the shortest setup in the rhythmic test (rhythmic Fmax3) showed similar relative intra-session reliability (T1, ICC = 0.898; T2, ICC > 0.755) compared to the dynamic test (dynamic Fmax3), intra-session differences during T2 were significant (p = 0.003) with low relative inter-session reliability [ICC = 0.334 (CI 95 % −0.033 to 0.620)].

**Table 2:** Relative and absolute reliability on dynamic and rhythmic weight bearing capacity in non-ambulatory patients with subacute stroke

	Intra-session reliability (T1)					Intra-session reliability (T2)					Inter-session reliability (T1/T2)					
	Mean ± SD	ICC (CI 95 %)	SEM	MDD	p^‡^	Mean ± SD	ICC (CI 95 %)	SEM	MDD	p^‡^	MD	SDD	ICC (CI 95 %)	SEM	MDD	p^§^
Dynamic																
Fmax15	75.0 ± 11.5	0.776 (0.642 to 0.895)	5.5	15.1	0.345	76.9 ± 9.7	0.618 (0.440 to 0.814)^†^	6.0	16.6	0.964	4.4	3.4	0.866 (0.732 to 0.935)	1.2	3.5	0.090
Fmax10	74.8 ± 11.5	0.795 (0.684 to 0.891)	5.2	14.4	0.260	76.9 ± 9.5	0.704 (0.576 to 0.828)^†^	5.2	14.3	0.370	4.8	4.5	0.812 (0.639 to 0.907)	1.9	5.4	0.054
Fmax5	74.0 ± 11.8	0.820 (0.720 to 0.899)	5.0	13.8	0.814	76.7 ± 9.6	0.830 (0.736 to 0.904)	3.9	10.9	0.112	5.6	4.9	0.769 (0.559 to 0.885)	2.4	6.6	0.041*
Fmax4	73.8 ± 11.2	0.840 (0.743 to 0.911)	4.5	12.4	0.687	76.4 ± 9.7	0.837 (0.740 to 0.909)	3.9	10.9	0.095	5.5	4.9	0.766 (0.558 to 0.883)	2.4	6.5	0.041*
Fmax3	73.5 ± 11.2	0.843 (0.735 to 0.915)	4.5	12.4	0.792	76.2 ± 10.0	0.829 (0.715 to 0.908)	4.1	11.5	0.067	5.5	5.5	0.740 (0.517 to 0.868)	2.8	7.8	0.102
Rhythmic																
Fmax15	74.4 ± 12.6	0.823 (0.740 to 0.897)	5.3	14.7	0.971	76.9 ± 11.1	0.735 (0.625 to 0.840)^†^	5.7	15.8	0.159	4.4	3.6	0.887 (0.746 to 0.948)	1.2	3.4	0.017*
Fmax10	74.5 ± 12.7	0.886 (0.825 to 0.935)	4.3	11.9	0.710	76.7 ± 11.1	0.684 (0.561 to 0.805)^†^	6.2	17.3	0.023*	4.8	3.5	0.852 (0.704 to 0.928)	1.3	3.7	0.043*
Fmax5	74.7 ± 13.0	0.891 (0.825 to 0.940)	4.3	11.9	0.534	76.0 ± 12.1	0.765 (0.645 to 0.865)	5.9	16.3	0.006*	5.8	5.0	0.818 (0.650 to 0.910)	2.1	5.9	0.127
Fmax4	74.5 ± 13.4	0.892 (0.823 to 0.942)	4.4	12.2	0.334	75.7 ± 12.7	0.772 (0.645 to 0.871)	6.1	16.9	0.001*	6.2	5.5	0.802 (0.623 to 0.902)	2.5	6.8	0.133
Fmax3	74.1 ± 13.9	0.898 (0.825 to 0.946)	4.4	12.3	0.670	76.2 ± 12.3	0.755 (0.603 to 0.866)	6.1	16.8	0.003*	11.2	10.2	0.334 (−0.033 to 0.620)^†^	8.3	23.0	0.611

Mean ± SD, SEM and MDD values are given in % body weight

*T* trial, *SD* standard deviation, *ICC* intra-class correlation coefficient, *CI* confidence interval, *SEM* standard error of the measurement, *MDD* minimal detectable difference, *p* probability, *MD* mean difference, *SDD* difference of the standard deviation, *Fmax* maximal force during a number of repetitions

* p < 0.05

^†^ICC < 0.740

^‡^Friedman test

^§^Wilcoxon test

## Discussion

A fast and easy-to-perform assessment to evaluate WBC in non-ambulatory patients with subacute stroke was developed and evaluated in order to provide a practical tool for clinical decision-making and efficacy analyses. The assessment can discriminate between patients with stroke and healthy controls and was shown to be highly reliable. It can be applied in a variety of clinical settings, and could be used to evaluate WBC in severely impaired patients with limited standing ability within the rehabilitation routine. In particular, patients early after stroke with severe hemiparesis suffer from de-conditioning and limited motor control, which restricts extended assessment of physical function. The introduction of dynamic repetitions within a short period of time to evaluate WBC is therefore of high importance for this population.

Previous studies have reported high intra-session reliability for forward and lateral weight shifting [2], good intra-session reliability for the body centre of mass displacement when transferring weight laterally into a single-leg posture [6], and high inter-session reliability over 2 separate days for WBC during different standing tasks [3]. In addition to the similar relative and absolute reliability results compared to previous research, this is the first study that condenses the assessment protocol into a single evaluation making it applicable for clinical routine. Although rhythmic facilitation during WBC was less reliable in this study, it presents an option to control execution speed during weight shifting/walking tasks, as a previous study has shown to increase effort in gait rehabilitation with acute stroke patients [11].

In contrast to previous research on weight distribution among patients with stroke [12, 13], this study found no significant between-group differences in weight bearing capacity in the static condition (also described as preferred weight distribution). This might be due to the instruction during the trials, which was: “to stand as still and balanced as possible” for 60 s. It must be hypothesised that avoidance of this instruction might have led to significant differences in weight distribution between patients with subacute stroke and healthy controls.

The present study has some limitations. A foot pressure plate has been used to evaluate force–time curves rather than a force plate, considered as the gold standard for weight distribution analyses. However, this study aimed to develop an assessment for routine clinical usage, which justifies the usage of a low-cost measurement device. The eligibility criteria on standing function limit the external validity of the proposed assessment. The instructions permitted participants to perform the movement in a comfortable way, rather than constraining the task with more specific instructions (e.g., standardise lateral hip and trunk displacement). The sample size was rather small to generalise the findings and propose recommendations for clinical practice. Future investigations will obtain longitudinal data with a larger sample to establish the usability and the predictability of the measurement. Furthermore, data on concurrent validity are of high importance, and the gold standard to rapidly assess WBC in a clinical environment needs to be determined. Other variables such as awareness, attention, and coordination that influence WBC might also be taken into account [14, 15].

In conclusion, 3 dynamic repetitions of loading the hemiparetic leg on a foot pressure plate are sufficient to assess WBC in non-ambulatory patients with subacute stroke. This is an important finding regarding the development and implementation of a fast and easy-to-perform assessment for routine clinical usage in patients with limited standing ability.

## Abbreviations

WBC: Weight-bearing capacity
BW: Body weight
ICC: Intra-class correlation
SEM: Standard error of measurement
MDD: Minimal detectable difference
CI: Confidence interval
